# Structural Evolution and Properties of Praseodymium Antimony Oxochlorides Based on a Chain-like Tertiary Building Unit

**DOI:** 10.3390/molecules28062725

**Published:** 2023-03-17

**Authors:** Wei-Yang Wen, Bing Hu, Tian-Yu Pan, Zi-Wei Li, Qian-Qian Hu, Xiao-Ying Huang

**Affiliations:** 1 College of Chemistry, Fuzhou University, Fuzhou 350108, China; 2 State Key Laboratory of Structural Chemistry, Fujian Institute of Research on the Structure of Matter, Chinese Academy of Sciences, Fuzhou 350002, China; 3 University of Chinese Academy of Sciences, Beijing 100049, China

**Keywords:** structural evolution, metal oxochloride, lanthanide antimony cluster, single-crystal structure, tertiary building unit, photodegradation

## Abstract

Unveiling the structural evolution of single-crystalline compounds based on certain building units may help greatly in guiding the design of complex structures. Herein, a series of praseodymium antimony oxohalide crystals have been isolated under solvothermal conditions via adjusting the solvents used, that is, [HN(CH_2_CH_3_)_3_][Fe^II^(2,2′-bpy)_3_][Pr_4_Sb_12_O_18_Cl_15_]·EtOH (**1**) (2,2′-bpy = 2,2′-bipyridine), [HN(CH_2_CH_3_)_3_][Fe^II^(2,2′-bpy)_3_]_2_[Pr_4_Sb_12_O_18_Cl_14_)_2_Cl]·N(CH_2_CH_3_)_3_·2H_2_O (**2**), and (H_3_O)[Pr_4_Sb_12_O_18_Cl_12.5_(TEOA)_0.5_]·2.5EtOH (**3**) (TEOA = mono-deprotonated triethanolamine anion). Single-crystal X-ray diffraction analysis revealed that all the three structures feature an anionic *zig-zag* chain of [Pr_4_Sb_12_O_18_Cl_15−*x*_]*_n_* as the tertiary building unit (TBU), which is formed by interconnections of praseodymium antimony oxochloride clusters (denoted as {Pr_4_Sb_12_}) as secondary building units. Interestingly, different arrangements or linkages of chain-like TBUs result in one-dimensional, two-dimensional layered, and three-dimensional structures of **1**, **2**, and **3**, respectively, thus demonstrating clearly the structural evolution of metal oxohalide crystals. The title compounds have been characterized by elemental analysis, powder X-ray diffraction, thermogravimetric analysis, and UV-Vis spectroscopy, and the photodegradation for methyl blue in an aqueous solution of compound **1** has been preliminarily studied. This work offers a way to deeply understand the assembly process of intricate lanthanide-antimony(III) oxohalide structures at the atomic level.

## 1. Introduction

Hierarchical assembly is an effective strategy for rational and precise construction of new structures [1,2,3,4,5,6,7]. Building units (BUs) are essential in the hierarchical assembly. Therefore, it is important to search for new secondary building units (SBUs), and even tertiary building units (TBUs), for assembling complex structures. Meanwhile, systematical demonstration of structural evolution at the atomic level is meaningful to understand the assembly process and, therefore, help to design new intricate structures [8]; however, this remains a challenge because of the difficulty in obtaining precise or single-crystalline structures in each hierarchical level.

Metal–oxo cluster-based crystalline compounds have attracted increasing attention for their intriguing structural characteristics and properties [9,10,11,12,13,14,15]. Various types of metal–oxo clusters with tunable composition, size, and symmetry, such as polyoxometalate cluster [16,17,18], 3d–4f oxide cluster [19,20], and lanthanide oxide cluster [21,22], have been developed as SBUs to construct cluster-based crystals with complex structures. We have reported the first discrete lanthanide antimony nanocluster of [Pr_4_Sb_12_O_18_Cl_17_]^5−^ (denoted as {Pr_4_Sb_12_}) with *T*_d_ symmetry and 12 terminally coordinated Cl^−^ ions as antennas in the cluster [23]. Subsequently, by changing the reaction conditions and introducing organic carboxylic acid ligands as bridging linkers to replace the terminally coordinated Cl^−^ ions, the {Pr_4_Sb_12_} clusters could act perfectly as SBUs to assemble diversiform compounds with one-dimensional (1-D) helical chain-like, ribbon-like, or tubular structure; two-dimensional (2-D) layered structure; and three-dimensional (3-D) interspersed or porous structure [24,25,26]. Very recently, we have isolated two giant superclusters in the form of the Rubik’s cube by eight-aggregation of {Pr_4_Sb_12_} clusters as SBUs, and the superclusters further acted as TBUs to construct a series of superstructures [8]. Importantly, we were able to isolate two intermediate superstructures with coexisting SBUs and TBUs in distinct single-crystalline form, which helped in revealing the structural evolution of superstructures at the atomic level. Of note, in these superstructures, some of the terminal Cl^−^ ion in {Pr_4_Sb_12_} clusters turn to function as bridging ligands, which accounts partially for the formation of the supercluster TBUs and superstructures with a high-dimensionality structure. This is not surprising, as the majority of metal oxyhalides are not 0-D structures, where halogen ions are bound to metal ions in bridging fashions, thus forming high-dimensionality structures [27,28,29,30,31]. Therefore, it is anticipated that, by manipulating the linkage modes of the surface Cl^−^ ions of the {Pr_4_Sb_12_} cluster, complex cluster-based structures with higher dimensionality may be obtained.

Herein, we report on a series of single-crystalline praseodymium antimony oxochloride compounds isolated under solvothermal conditions in the presence of different solvents, including [HN(CH_2_CH_3_)_3_][Fe^II^(2,2′-bpy)_3_][Pr_4_Sb_12_O_18_Cl_15_]·EtOH (**1**) (2,2′-bpy = 2,2′-bipyridine), [HN(CH_2_CH_3_)_3_][Fe^II^(2,2′-bpy)_3_]_2_[Pr_4_Sb_12_O_18_Cl_14_)_2_Cl]·N(CH_2_CH_3_)_3_·2H_2_O (**2**), and (H_3_O)[Pr_4_Sb_12_O_18_Cl_12.5_(TEOA)_0.5_]·2.5EtOH (**3**) (TEOA = mono-deprotonated triethanolamine). The compounds have been characterized by single-crystal X-ray diffraction (SCXRD), elemental analysis (EA), powder X-ray diffraction (PXRD), thermogravimetric analysis (TGA), and UV-Vis spectroscopy. SCXRD analysis revealed that the title crystals are characterized by *zig-zag*, chain-like [Pr_4_Sb_12_O_18_Cl_15−*x*_]*_n_* TBUs constructed by interconnecting {Pr_4_Sb_12_} cluster SBUs via the edge-sharing of two Cl ions. The distinct assemblies of these TBUs resulted in the 1-D, 2-D, and 3-D structures of **1**, **2**, and **3**, respectively. Preliminary photocatalytic study indicated that compound **1** exhibited photodegradation for methyl blue in an aqueous solution. This work may provide certain guidance for preparing intricate single-crystalline compounds based on unique metal oxohalide cluster-based BUs. 

## 2. Results and Discussions

### 2.1. Crystal Structure Descriptions

Single crystals of the four title compounds were obtained via the solvothermal method. Single-crystal X-ray diffraction analysis was performed for compounds **1**, **2**, and **3**. The crystallographic data and structural refinement details for **1**, **2**, and **3** are depicted in Table S1. 

Crystal data for [HN(CH_2_CH_3_)_3_][Fe^II^(2,2′-bpy)_3_][Pr_4_Sb_12_O_18_Cl_15_]·EtOH (**1,** C_38_H_46_O_19_N_7_Cl_15_FeSb_12_Pr_4_, *M* = 3517.19 g/mol): monoclinic, space group *P*2_1_/*c* (no. 14); *a* = 17.3764(10) Å; *b* = 15.6415(9) Å; *c* = 30.6147(18) Å; *β* = 105.791(6)°; *V* = 8006.8(8) Å^3^; *Z* = 4; *T* = 298(2) K; *μ*(Mo*Kα*) = 7.090 mm^−1^; *D*_calc_ = 2.918 g/cm^3^; 79,381 reflections measured (4.814° ≤ 2*θ* ≤ 54.656°); 16,255 unique (*R*_int_ = 0.0609, *R*_sigma_ = 0.0518), which were used in all calculations. The final *R*_1_ was 0.0345 (*I* > 2*σ*(*I*)), and *wR*_2_ was 0.0717 (all data). 

Crystal data for [HN(CH_2_CH_3_)_3_][Fe^II^(2,2′-bpy)_3_]_2_[Pr_4_Sb_12_O_18_Cl_14_)_2_Cl]·N(CH_2_CH_3_)_3_·2H_2_O (**2**, C_72_H_83_O_38_N_14_Cl_29_Fe_2_Sb_24_Pr_8_, *M* = 6941.82 g/mol): orthorhombic; space group *Pccn* (no. 56); *a* = 15.1348(5) Å; *b* = 31.4569(7) Å; *c* = 32.3294(9) Å; *V* = 15391.8(7) Å^3^; *Z* = 4; *T* = 298.5(2) K; *μ*(Cu*Kα*) = 58.396 mm^−1^; *D*_calc_ = 2.996 g/cm^3^; 75,286 reflections measured (5.354° ≤ 2*θ* ≤ 150.812°); 13,605 unique (*R*_int_ = 0.1299, *R*_sigma_ = 0.0768), which were used in all calculations. The final *R*_1_ was 0.0647 (*I* > 2*σ*(*I*)), and *wR*_2_ was 0.1977 (all data).

Crystal data for (H_3_O)[Pr_4_Sb_12_O_18_Cl_12.5_(TEOA)_0.5_]·2.5EtOH (**3,** C_8_H_25_O_23_N_0.5_Cl_12.5_Sb_12_Pr_4_, *M* = 2964.18 g/mol): monoclinic; space group *C*2/*m* (no. 12); *a* = 30.385(2) Å; *b* = 14.9201(12) Å; *c* = 11.2014(7) Å; *β* = 90.379(7)°; *V* = 5078.1(6) Å^3^; *Z* = 4; *T* = 295(2) K; *μ*(Mo*Kα*) = 10.743 mm^−1^; *D*_calc_ = 3.877 g/cm^3^; 13,723 reflections measured (4.820° ≤ 2*θ* ≤ 58.366°); 6374 unique (*R*_int_ = 0.0340, *R*_sigma_ = 0.0525), which were used in all calculations. The final *R*_1_ was 0.0330 (*I* > 2*σ*(*I*)), and *wR*_2_ was 0.0734 (all data).

**Figure 1:**
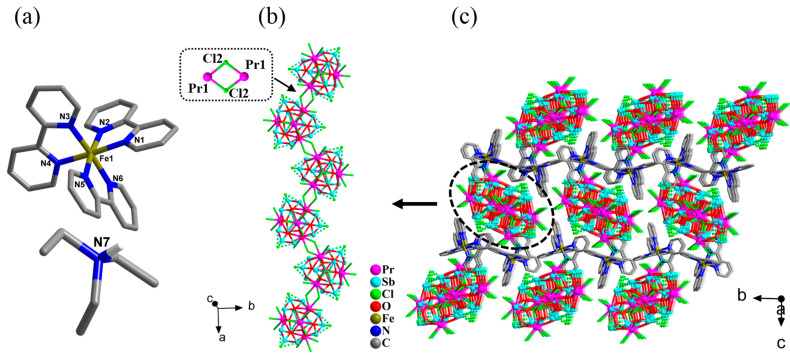
(**a**) Structure of the cation [Fe(2,2′-bpy)_3_]^2+^ and protonated triethylamine present in compound **1**. (**b**) View of one 1-D chain-like anion of [Pr_4_Sb_12_O_18_Cl_15_]*_n_* extended along the *a*-axis in compound **1**; a [Pr-Cl_2_-Pr] unit is highlighted with atomic labeling. (**c**) View along the *a*-axis of the packing of the anionic 1-D chains together with the [Fe(2,2′-bpy)_3_]^2+^ cations in compound **1**. For clarity, the protonated triethylamine, lattice ethanol, and H atoms are omitted.

The asymmetric unit of the compound [HN(CH_2_CH_3_)_3_][Fe(2,2′-bpy)_3_][Pr_4_Sb_12_O_18_Cl_15_]·EtOH (**1**) contains one anionic [Pr_4_Sb_12_O_18_Cl_15_]^3−^ cluster, one cationic [Fe^II^(2,2′-bpy)_3_]^2+^ complex, and one [HN(CH_2_CH_3_)_3_]^+^ cation, as well as one lattice ethanol molecule. Each cluster connects to two neighboring ones via each of the two edge-sharing Cl atoms forming a [Pr-Cl_2_-Pr] four-membered ring to form a *zig-zag* chain of [Pr_4_Sb_12_O_18_Cl_15_]*_n_* along the *a*-axis. The counterions of the [Fe^II^(2,2′-bpy)_3_]^2+^ complexes and protonated triethylamine, as well as the lattice solvent molecules, are located in between the chains (Figure 1 and Figure S1 in Supplementary Materials). Compared with the original isolated {Pr_4_Sb_12_} cluster, four terminal Cl ions from two Pr^3+^ ions are changed into the bridging mode in each cluster of **1**, thus resulting in a 1-D chain.

**Figure 2:**
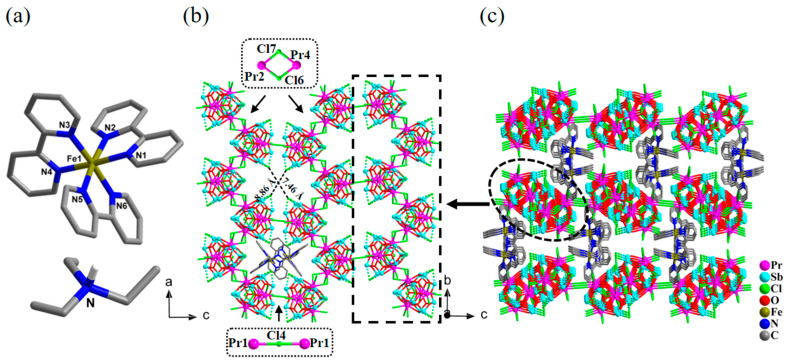
(**a**) Structure of the cations [Fe(2,2′-bpy)_3_]^2+^ and protonated triethylamine in compound **2**. (**b**) View of one of the 2-D layers in compound **2** along the *b*-axis; a couple of [Fe(2,2′-bpy)_3_]^2+^ complex cations located up and down the window within the layer are shown, and the interconnecting units of [Pr-Cl_2_-Pr] and [Pr-Cl-Pr] are highlighted. (**c**) Packing diagram for compound **2** viewed along the *b*-axis. For clarity, the protonated triethylamine, lattice water molecules and triethylamine molecules, and H atoms in [Fe(2,2′-bpy)_3_]^2+^ cations are not shown.

The asymmetric unit of the compound [HN(CH_2_CH_3_)_3_][Fe^II^(2,2′-bpy)_3_]_2_[Pr_4_Sb_12_O_18_Cl_14_)_2_Cl]·N(CH_2_CH_3_)_3_·2H_2_O (**2**) contains half of a formular unit, that is, one anionic [Pr_4_Sb_12_O_18_Cl_14.5_]^2.5−^ cluster, one cationic [Fe^II^(2,2′-bpy)_3_]^2+^ complex, and half of a protonated triethylamine cation, as well as half of a neutral triethylamine and one water molecule. The two types of cations in **2** are illustrated in Figure 2a. Similar to that in compound **1**, there are also 1-D *zig-zag* chains of [Pr_4_Sb_12_O_18_Cl_15_]*_n_* present in compound **2** that are extended along the *a*-axis, as highlighted in Figure 2b. However, the chains in **2** are further interlinked to each other via sharing a single chloride bridge to result in 2-D layers along the *ac* plane (Figure 2b). The layered structure in **2** can be regarded as a further aggregation of the isolated chains in structure **1** as TBUs. Compared to the original isolated {Pr_4_Sb_12_} cluster, five terminal Cl ions from three Pr^3+^ ions are changed into the bridging mode in each cluster of **2**, and each {Pr_4_Sb_12_} cluster connects to three neighboring ones to construct the 2-D network with windows with a size of 7.46 × 8.86 Å^2^. The layers then further pack along the *b*-axis in a staggered form (Figure 2c and Figure S2); the guest molecules, as well as [Fe^II^(2,2′-bpy)_3_]^2+^ complex groups and protonated triethylamine, are located between layers.

**Figure 3:**
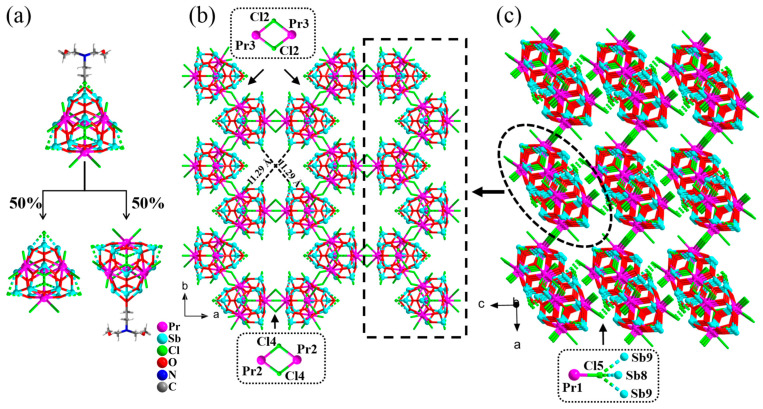
(**a**) Two possible anionic structure types with equal probability in **3**. (**b**) The porous 3-D framework structure of compound **3** viewed along the *c*-axis; one of the chains and the connecting [Pr-Cl_2_-Pr] units are highlighted. (**c**) The 3-D framework of **3** viewed along the *b*-axis. For clarity, the hydrated proton and lattice ethanol molecules are not shown.

The asymmetric unit of the compound (H_3_O)[Pr_4_Sb_12_O_18_Cl_12.5_(TEOA)_0.5_]·2.5EtOH (**3**) consists of half a formula unit. The position of one cap in the anion cluster is co-occupied by the Cl^−^ ion and the deprotonated triethanolamine anion in a statistically distributed manner with an occupancy of 0.5/0.5 (Figure 3a), endowing compound **3** with two types of anionic structures; that is, the structural formula of the anion cluster is [Pr_4_Sb_12_O_18_Cl_13_]^−^ when the position of the cap is a Cl^−^ ion and is [Pr_4_Sb_12_O_18_Cl_12_(TEOA)]^−^ when the cap is an O from the deprotonated triethanolamine. Also similar to the chain in structure **1**, the clusters connect to each other via sharing a Cl_2_ bridge (Cl(3)_2_) along the *b*-axis into a chain (Figure 3b); meanwhile, the chains further link to each other along the *a*-axis through another set of Cl_2_ bridges (Cl(4)_2_) to form a layer extended along the *ab* plane (Figure 3b). Interchain linking via sharing a Cl bridge (Cl(5)) between a Pr atom of the {Pr_4_Sb_12_} cluster in one layer and a (Sb_3_O_3_) unit of the {Pr_4_Sb_12_} cluster from another layer finally creates a 3-D porous framework, with a channel of 11.29 × 11.29 Å^2^ along the *c*-axis (Figure 3b,c). The connection of Pr-Cl-Sb_3_O_3_ is firstly found in the {Pr_4_Sb_12_} cluster-based structures [23,24,25,26]. Hydrated protons act as cations, and as well as ethanol solvent molecules, are located in the 1-D channels along the *c*-axis. Compared to the original isolated {Pr_4_Sb_12_} cluster [23], seven terminal Cl ions from all four Pr^3+^ ions in each {Pr_4_Sb_12_} cluster of **3** are changed into the bridging mode, and each cluster connects to four neighboring ones to construct the 3-D framework. On the other hand, structure **3** can also be regarded as a 3-D framework that is directly constructed by the interconnection of the chain-like TBUs that are also present in compounds **1** and **2**.

**Figure 4:**
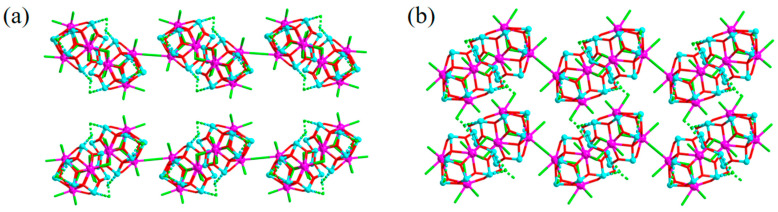
(**a**) Illustration of the stacking of two adjacent layers in compound **2**, indicating that the Cl^−^ ion capped by Sb_3_O_3_ in one layer is far from the Pr^3+^ ion in the adjacent layer. (**b**) Illustration of adjacent layers in compound **3** that are interconnected by the bonding between a Pr^3+^ ion in one layer with the Cl^−^ ion capped by Sb_3_O_3_ in the adjacent layer.

When comparing the structures of **2** and **3**, we found that the same chain TBUs aggregate into the 2-D layer in compound **2** and the 3-D framework in compound **3**, respectively, which corresponds to different connecting modes of the cluster-based chain TBUs in the structural assembly. Compared to the two sets of Pr-Cl_2_-Pr and one set of Pr-Cl-Pr bridges in **2**, there is an extra connection of Pr-Cl-Sb_3_O_3_ from the last Pr^3+^ ion to result in the 3-D framework of **3** rather than the 2-D network in **2** (Figure 4). Relatively speaking, the smaller cation of (H_3_O)^+^ in structure **3** may help to induce a structure with a higher dimension than the larger ones of [Fe(2,2′-bpy)_3_]^2+^ and triethylamine in structures **1** and **2**.

**Figure 5:**
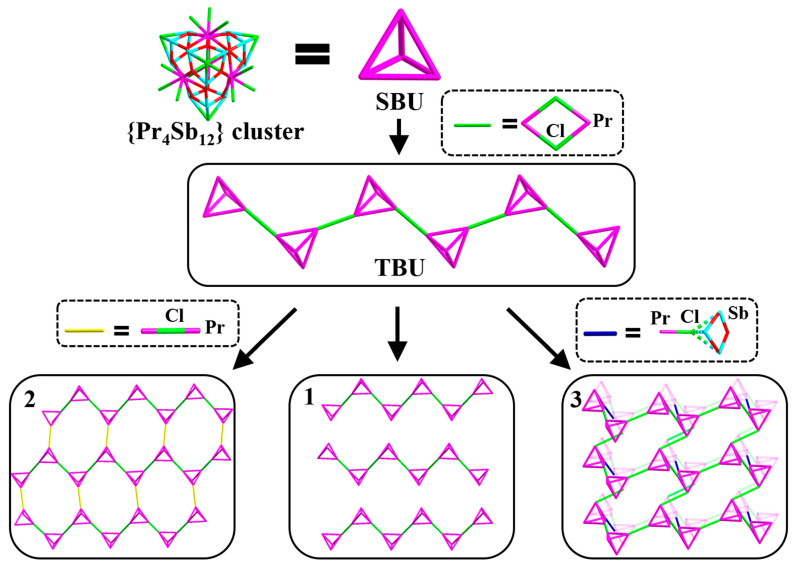
Schematic diagram showing the structural evolution process of the chain-like TBUs-based title structures.

As a whole, there are obvious relationships of structures among the three title compounds. The {Pr_4_Sb_12_} clusters act as SBUs to aggregate into a *zig-zag* chain, which then acts as a TBU to assemble the title compounds with distinct structural dimensionalities, that is, 1-D for **1**, 2-D for **2**, and 3-D for **3**. As above-mentioned, the dimensionalities and hierarchy level of the cluster-based title structures are controlled by the numbers and fashions of linkage units that interconnect the 1-D chain-like TBUs. Without additional linkers, isolated 1-D chain-like anions are evidenced in **1**, while additional Pr-Cl-Pr bridges in **2** and Pr-Cl_2_-Pr, as well as unique Pr-Cl-Sb_3_O_3_ bridges in **3**, lead to 2-D and 3-D TBU-based networks, respectively. Unlike the previously reported {Pr_4_Sb_12_} cluster–organic structures [24,25,26], in which the linkers are bi/tri functional organic ligands, herein, the title structures are formed through interconnections of TBUs by inorganic linkage units, such as Pr-Cl-Pr and Pr-Cl-Sb_3_O_3_. This is also different from the case in the assembly of single-crystalline superstructures we just reported, in which the (FeCN_6_) and/or the Ac (acetate group) function as linkers along with Cl^−^ to realize the aggregation of eight {Pr_4_Sb_12_} clusters into supercluster TBUs rather than chain-like TBUs in this work. Overall, by analyzing the crystal structure motifs in detail, a clear structural evolution of the cluster-based structures is achieved (Figure 5).

### 2.2. The Basic Characterizations

**Figure 6:**
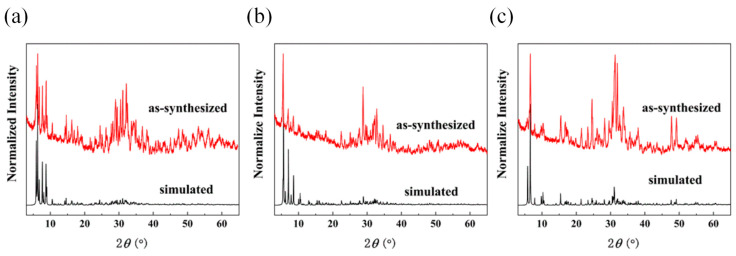
Comparison of powder X-ray diffractograms of title compounds **1** (**a**), **2** (**b**) and **3** (**c**) with that simulated from corresponding single-crystal X-ray diffraction data.

**Figure 7:**
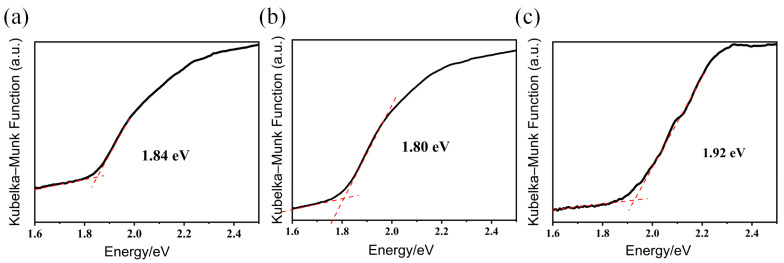
Solid-state UV-Vis absorption spectra of title compounds **1** (**a**), **2** (**b**) and **3** (**c**).

The high purity of the title compounds was well demonstrated by the fact that the experimental PXRD pattern matched well with the calculated results from the single-crystal X-ray diffraction data (Figure 6), along with various characterizations, such as elemental analysis (EA) and TGA. Note that the PXRD patterns feature broad and low-intensity diffraction peaks, which are common for these types of high-nuclearity cluster-based compounds [8,23,24,25,26]. The TGA for compounds **1** and **3** was carried out under a nitrogen atmosphere in the temperature range of 20–1000 °C (Figure S3). The UV-Vis absorption spectra of compounds **1**, **2**, and **3** were collected to characterize their optical absorption abilities and bandgaps. As shown in Figure 7, the optical absorption properties of the title compounds are in accordance with the colors of the compounds in each case, and the bandgaps are calculated to be 1.84, 1.80, and 1.92 eV for compounds **1**, **2**, and **3**, respectively.

### 2.3. The Photodegradation Characterizations of Compound ***1***

**Figure 8:**
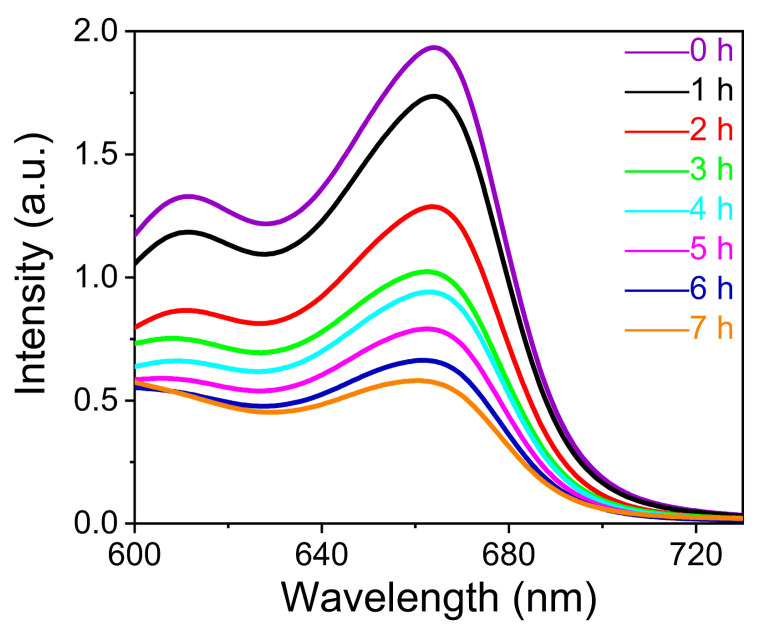
Liquid UV absorption spectra of compound **1** tested after different illumination time periods of photodegradation of MB solutions under visible light irradiation (initial solubility of ~10 ppm, *V*/*m* = 1000 mL/g, experimental temperature of 20 °C). Inset picture shows the change in color of the solution before and after degradation.

In recent years, a number of scholars have used metal–oxo cluster-based compounds to study the performance of the adsorption and/or degradation of dyes [32,33,34,35]. In this work, compound **1** was chosen as the representative to study the photodegradation property of these cluster-based compounds. In determining the stability of compound **1**, it was found that it can dissolve completely in water to form a red, clear solution (Figure S4). In view of this, we investigated the photocatalytic performance of compound **1** as a homogeneous reaction catalyst for the photocatalytic degradation of MB solutions. The absorbance of the MB solution was measured by UV-Vis spectrophotometer after different illumination times. It was found that the absorption intensity of the MB solution at the maximum absorption wavelength of 668 nm decreased with the illumination duration, illustrating that compound **1** has an obvious degradation ability towards MB. After illumination of 7 h, the degradation of MB by compound **1** was close to 71.8% (Figure 8).

## 3. Materials and Methods

All reagents for synthesis were purchased from commercial sources and used without further purification. The detailed information for the reagents is listed as follows: praseodymium acetate pentahydrate (Pr(Ac)_3_·5H_2_O, 99%, Tianjin Yingda Rare and Expensive Chemical Reagent Co., Ltd., Tianjin, China); antimony trichloride (SbCl_3_, 99%, Beijing Hwrkchemical Co,. Ltd., Beijing, China); sodium ferrocyanide decahydrate (Na_4_Fe(CN)_6_·10H_2_O, 98%, Tianjin Guangfu Fine Chemical Research Institute, Tianjin, China); 2,2′-bipyridine (C_10_H_8_N_2_, 99%, Beijing Hwrkchemical Co,. Ltd., Beijing, China); triethanolamine (C_6_H_15_NO_3_, 98%, Sinopharm Chemical Reagent Co., Ltd., Shanghai, China); triethylamine (C_6_H_15_N, 98%, Sinopharm Chemical Reagent Co., Ltd., Shanghai, China); ethanol (C_2_H_6_O, 98%, Greagent, Shanghai Titan Technology Co., Ltd., Shanghai, China).

**Preparation of 1.** A mixture of Pr(Ac)_3_·5H_2_O (0.3602 g, 0.88 mmol), SbCl_3_ (0.5985 g, 2.625 mmol), Na_4_Fe(CN)_6_·10H_2_O (0.1089 g, 0.225 mmol), and 2,2′-bpy (0.0674 g, 0.43 mmol) in TEOA/TEA/ethanol (0.2 mL/0.4 mL/9 mL) was sealed in a 28 mL Teflon-lined stainless-steel autoclave at 150 °C for 4.25 days, then cooled to room temperature (RT). The product was washed several times with anhydrous ethanol and then filtered; the solid sample was then dried in air naturally. The resultant solid consisted of a large portion of indefinite powder and a small portion of targeted crystals. Dark-red block-like crystals of compound **1** (yield: 110.4 mg, 11.1% based on Pr) were picked up by manual selection. Elemental analysis calculated—(%) for C_38_H_46_O_19_N_7_Cl_15_FeSb_12_Pr_4_: C 12.97, H 2.79, N 1.32; found: C 12.64, H 1.53, N 2.86.

**Preparation of 2.** A mixture of Pr(Ac)_3_·5H_2_O (0.3602 g, 0.88 mmol), SbCl_3_ (0.5985 g, 2.625 mmol), Na_4_Fe(CN)_6_·10H_2_O (0.1089 g, 0.225 mmol), and 2,2′-bpy (0.0674 g, 0.43 mmol) in TEOA/TEA/ethanol (0.2 mL/0.4 mL/9 mL) was sealed in a 28 mL Teflon-lined stainless-steel autoclave at 150 °C for 5 days, then cooled to RT. The product was washed several times with anhydrous ethanol and then filtered; the solid sample was then dried in air naturally. The resultant solid consisted of a large portion of indefinite powder and a small portion of targeted crystals. Dark-red, hexagonal, sheet-like crystals of compound **2** (yield: 22.8 mg, 2.32% based on Pr) were picked up by manual selection. Elemental analysis calculated—(%) for C_72_H_83_O_38_N_14_Cl_29_Fe_2_Sb_24_Pr_8_: C 12.46, H 1.21, N 2.82; found: C 12.39, H 1.33, N 3.07.

**Preparation of 3.** A mixture of Pr(Ac)_3_·5H_2_O (0.2044 g, 0.5 mmol), SbCl_3_ (0.4011 g, 1.75 mmol), Na_4_Fe(CN)_6_·10H_2_O (0.0726 g, 0.15 mmol), and 2,2′-bpy (0.0469 g, 0.3 mmol) in TEOA/TEA/H_2_O/ethanol (0.2 mL/0.4 mL/0.5 mL/5.5 mL) was sealed in a 20 mL Teflon-lined stainless-steel autoclave at 150 °C for 4.25 days, then cooled to RT. The product was washed several times with ultrasound in anhydrous ethanol and then filtered; the solid sample was then dried in air naturally. Some title crystals together with indefinite powder were found. Red block-like crystals of compound **3** (yield: 122.3 mg, 25.7% based on Pr) were picked up by manual selection. Elemental analysis calculated—(%) for C_8_H_25_O_23_N_0.5_Cl_12.5_Sb_12_Pr_4_: C 3.24, H 0.85, N 0.24; found: C 3.24, H 0.90, N < 0.3.

The {Pr_4_Sb_12_} cluster-based compounds previously reported by us were all synthesized by using 2-methylpyridine and water as solvents under solvothermal conditions [23,24,25,26]. The mixed-solvents strategy has proved to be effective in preparing new compounds [13,36]. Therefore, a mixture of TEOA, TEA, and ethanol was utilized as the solvent system to prepare the title compounds. Furthermore, in our early attempts to synthesize {Pr_4_Sb_12_} cluster-based compounds, various sources of iron, such as FeCl_3_·6H_2_O, Fe(NO_3_)_3_·9H_2_O, and Na_4_[Fe(CN)_6_]·10H_2_O, were used. However, the title compounds could only be obtained in a mixed-solvent system and by using Na_4_[Fe(CN)_6_]·10H_2_O as the iron source. Although many attempts were made, the yield of the syntheses was still low, indicating the complexity of the reactions.

**Characterizations**. Powder X-ray diffraction (PXRD) patterns were obtained on a Rigaku Miniflex-II diffractometer by using Cu K_α_ radiation (*λ* = 1.54178 Å) with an angular range of 2*θ* = 3° − 65° at 30 KV, 15 mA, and a step size of 0.2. The simulated PXRD pattern was calculated from the SCXRD data using the *Mercury* program. Elemental analyses (EAs) of C, H, and N were performed using a German Elementary Vario EL III instrument. Energy-dispersive spectroscopy (EDS) was obtained by a JEOL JSM-6700F scanning electron microscope. Thermogravimetric analyses (TGA) were performed using crystalline sample loads in Al_2_O_3_ crucibles with a NETZSCH STA 449F3 unit at a heating rate of 10 K min^−1^ under a N_2_ atmosphere and in the temperature range of 25–1000 °C (heating rate, 10 °C min^−1^). UV-Vis absorbance photometric tests were carried out on a UV-2600 and PerkinElmer Lambda 350 UV-Vis spectrophotometer at RT in the range of 200–800 nm. Solid-state optical diffuse reflectance spectra were recorded on a Shimadzu 2600 UV/vis spectrometer at RT in the range of 200–800 nm. A BaSO_4_ plate was utilized as a standard, which possesses 100% reflectance. The absorption data were then obtained from the reflectance spectra by using the Kubelka–Munk function *α*/*S* = (1 − *R*)^2^/2*R*, where *α* refers to the absorption coefficient, *S* refers to the scattering coefficient, and *R* refers to the reflectance.

**Photodegradation experiments.** A methylene blue (MB) solution at a concentration of 100 ppm was prepared and diluted into MB standard solutions of 10, 8, 6, 4, 2, and 0 ppm. The absorbance of different concentrations of MB solutions was measured at the maximum absorption wavelength of 668 nm. The standard working curve of methylene blue solution was established using concentration (*C*) as the horizontal coordinate and absorbance (*A*) as the vertical coordinate (Figure S4). Then, 100 mg of compound **1** was ground into a photocatalytic reactor containing 100 mL of methylene blue solution (10 ppm) and stirred for 30 min in the dark to reach the adsorption–desorption equilibrium. A sample was taken as a standard, and then the absorbance of the sample at 668 nm was determined spectrophotometrically at 1 h intervals under light conditions using a 0.22 μm pinhead filter, and the concentration of the sample was calculated as *C*_t_. The calculation formula is as follows: *D* = (*C*_0_ − *C*_t_)/*C*_0_ × 100%. *C*_0_ is the concentration of MB in the mixed system at the initial equilibrium, and *C*_t_ is the concentration of MB in the system at various times of the reaction.

**Single-Crystal X-ray Diffraction (SCXRD).** Crystals of suitable size were selected for immersion in crystal oil under an optical microscope, and then a suitable glass wire was selected to hold the crystals on top of the glass wire for SCXRD characterization. The single-crystal X-ray diffraction data for **1** and **3** were collected on a SuperNova CCD diffractometer with graphite monochromatic Mo K_α_ radiation (*λ* = 0.71073 Å) at 295 K. The single-crystal X-ray diffraction data for **2** were collected on a SuperNova CCD diffractometer with graphite monochromatic Cu K_α_ radiation (*λ* = 1.54178 Å) at 298 K. The structures were solved by direct methods and refined by full-matrix least-squares on *F*^2^ using the SHELX-2018 program package [37]. In structure **1**, both [HN(CH_2_CH_3_)_3_]^+^ and lattice EtOH molecules are disordered over two positions, with a refined SOF ratio of ca. 0.75: 0.25. Accordingly, some soft restraints, such as SIMU, ISOR, and DELU, were applied to the obtained reasonable geometries and displacements for disordered atoms. Another feature is that one of the capping Cl ions coexists with the (TEOA)^−^ anion; their SOFs were refined to be close to 0.5 and 0.5, respectively, and finally were fixed at 0.5 and 0.5, respectively. The squeeze routine in the *PLATON* program was applied for structures **2** and **3** to squeeze out the cations (e.g., [HN(CH_2_CH_3_)_3_]^+^ and H_3_O^+^) and lattice molecules (i.e., EtOH, H_2_O, and N(CH_2_CH_3_)_3_) that could not be found from the difference-Fourier maps and/or refined properly [38]. Also of note is that, although many attempts to obtain high-quality crystals for compound 2 were made, the SCXRD data for **2** were always imperfect. However, the primary anionic structures of **2** were reliably determined. CCDC NO. 2241063 (for **1**), 2241064 (for **2**), and 2241065 (for **3**) contain the supplementary crystallographic data for this paper. These data can be obtained free of charge from The Cambridge Crystallographic Data Centre via www.ccdc.cam.ac.uk/data_request/cif (accessed on 18 November 2022).

## 4. Conclusions

In summary, we have constructed a chain-like TBU of [Pr_4_Sb_12_O_18_Cl_15−*x*_]*_n_* by aggregation of {Pr_4_Sb_12_} clusters. Through careful adjustment of the synthetic conditions, especially the solvents, three new structures based on the chain-like TBU have been obtained, including structure **1** featuring isolated 1-D anionic chains, structure **2** featuring an anionic 2-D layer, and structure **3** holding an anionic 3-D framework. A detailed analysis of the three title structures enabled us to clearly demonstrate at the atomical level the step-by-step evolution of this family of chain-like TBU-based complex structures, thus offering an excellent example for studying the precise design and controllable molecular assembly of new single-crystal cluster-based structures. The photodegradation ability of compound **1** was investigated preliminarily. Future study will focus on discovering new SBUs and TBUs towards the hierarchical assembly of single-crystal superstructures, especially on the basis of searching for new types of linkers for SBUs and TBUs.

## Data Availability

All the available data are incorporated in the MS.

## Supplementary Materials

The following supporting information can be downloaded at: https://www.mdpi.com/article/10.3390/molecules28062725/s1. Table S1: Crystallographic data and structural refinement details for the title compounds; Figure S1: View along the *b*-axis of the packing of anionic chains together with the [Fe(2,2′-bpy)_3_]^2+^ cations in compound **1**. For clarity, the guest molecules and H atoms are omitted; Figure S2: View along the *b*-axis of the packing of the anionic layers in compound **2**; Figure S3: Thermogravimetric test curves for compounds **1** (a) and **3** (b); Figure S4. Photograph for the solution formed by dissolving 4 mg compound **1** in 4 mL water; Figure S5: Standard curve for methylene blue solution; the horizontal coordinate is the concentration and the vertical coordinate is the absorbance.
