# Supplementary material for: Structural Evolution and Properties of Praseodymium Antimony Oxochlorides Based on a Chain-like Tertiary Building Unit
Source: Molecules. 2023 Mar 17;28(6):2725. doi: 10.3390/molecules28062725 (PMC10051633; doi:10.3390/molecules28062725)
Supplement: Supplementary file 1 [file molecules-28-02725-s001.zip › molecules-2243625-supplementary.pdf]

Table S1. Crystallographic data and structural refinement details for the title compounds.

	**1**	**2**	**3**
Empirical formula	$C_{38}H_{46}O_{19}N_{7}Cl_{15}FeSb_{12}Pr_{4}$	$C_{72}H_{83}O_{38}N_{14}Cl_{29}Fe_{2}Sb_{24}Pr_{8}$	$C_{8}H_{25}N_{0.5}Cl_{12.5}Sb_{12}Pr_{4}O_{23}$
Formula weight	3517.19	6941.82	2964.05
Crystal system	monoclinic	orthorhombic	monoclinic
Space group	$P2_1/c$	*Pccn*	$C2/m$
T/K	17.3764(10)	15.1348(5)	30.385(2)
λ/ Å	15.6415(9)	31.4569(7)	14.9201(12)
a/ Å	30.6147(18)	32.3294(9)	11.2014(7)
b/ Å	90	90	90
c/ Å	105.791(6)	90	90.379(7)
β/ º	90	90	90
V/ Å^3	8006.8(8)	15391.8(7)	5078.1(6)
Z	4	4	4
D_c/ Mg·m^{-3}	2.918	2.996	3.877
μ/mm^{-1}	7.090	58.396	10.743
F(000)	6416	12632	5284
Measured refls.	79381	75286	13723
Independent refls.	16255	13605	6374
R_{int}	0.0609	0.1299	0.0340
No. of parameters	956	772	300
GOF	1.068	1.053	1.051
$^aR_1$, $^bwR_2$ [$I > 2\sigma(I)$]	R_1 = 0.0345, wR_2 = 0.0605	R_1 = 0.0647, wR_2 = 0.1699	R_1 = 0.0330, wR_2 = 0.0659
$^aR_1$, $^bwR_2$ (all data)	R_1 = 0.0641, wR_2 = 0.0717	R_1 = 0.0916, wR_2 = 0.1977	R_1 = 0.0515, wR_2 = 0.0734

$^aR_1 = \sum\|F_o| - |F_c\|/\sum|F_o|$. $^bwR_2 = [\sum w(F_o^2 \quad F_c^2)^2/\sum w(F_o^2)^2]^{1/2}$

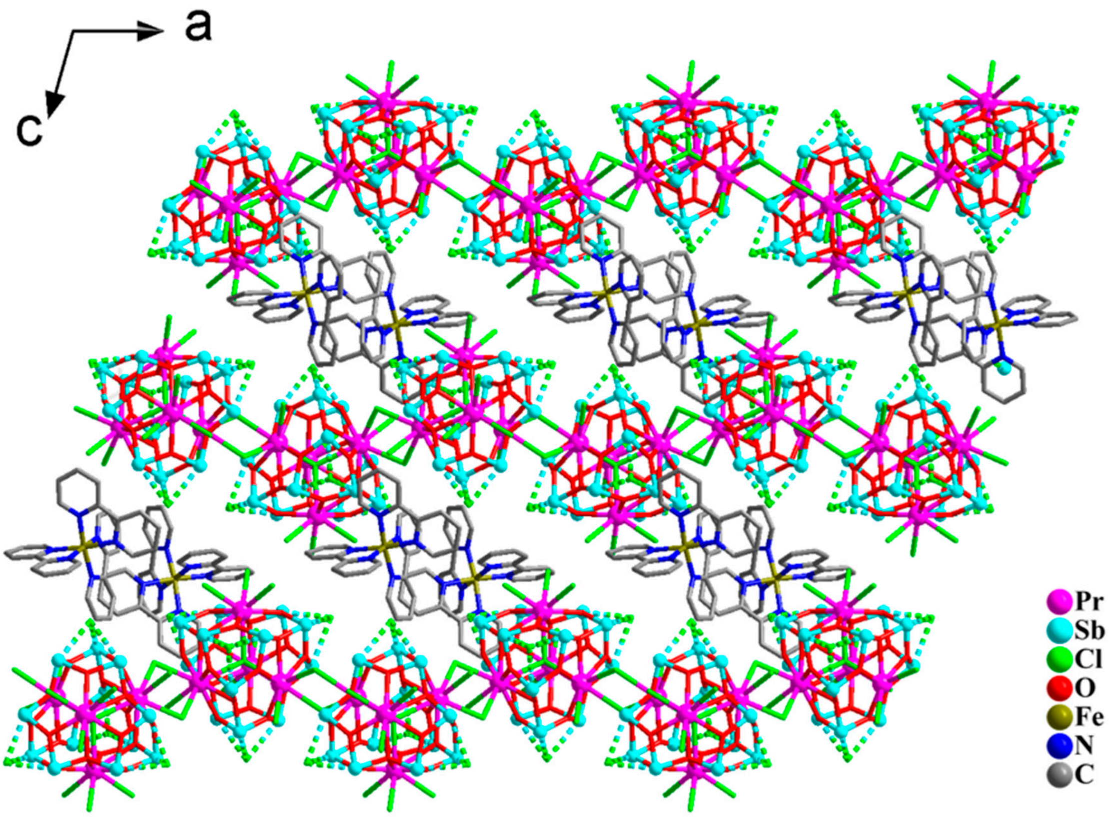

Figure S1. View along the *b*-axis of the packing of anionic chains together with the $[Fe(2,2'\text{-bpy})_3]^{2+}$ cations in compound **1**. For clarity, the guest molecules and H atoms are omitted.

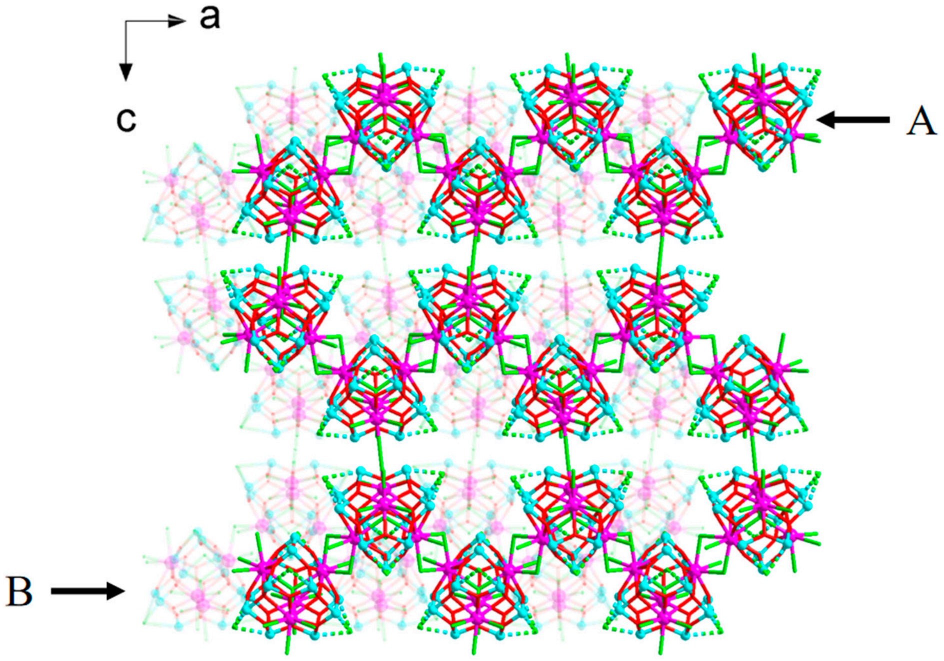

Figure S2. View along the *b*-axis of the packing of the anionic layers in compound **2**.

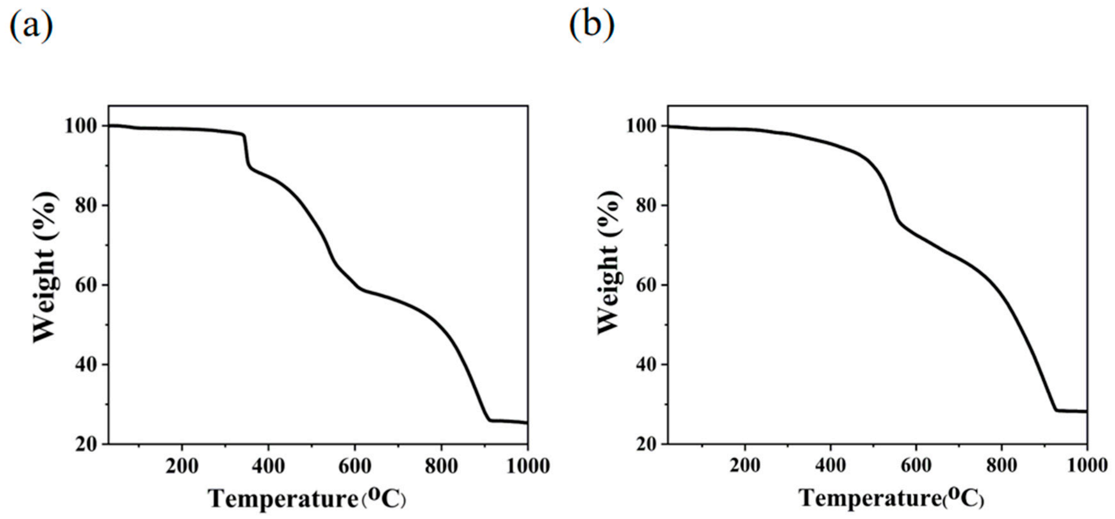

Figure S3. Thermogravimetric curves for compounds **1** (a) and **3** (b).

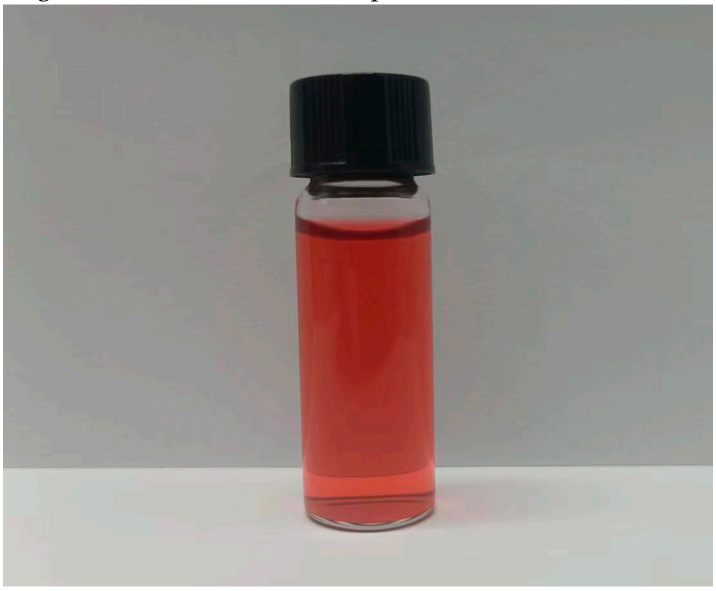

Figure S4. Photograph for the solution formed by dissolving 4 mg compound **1** in 4 mL water.

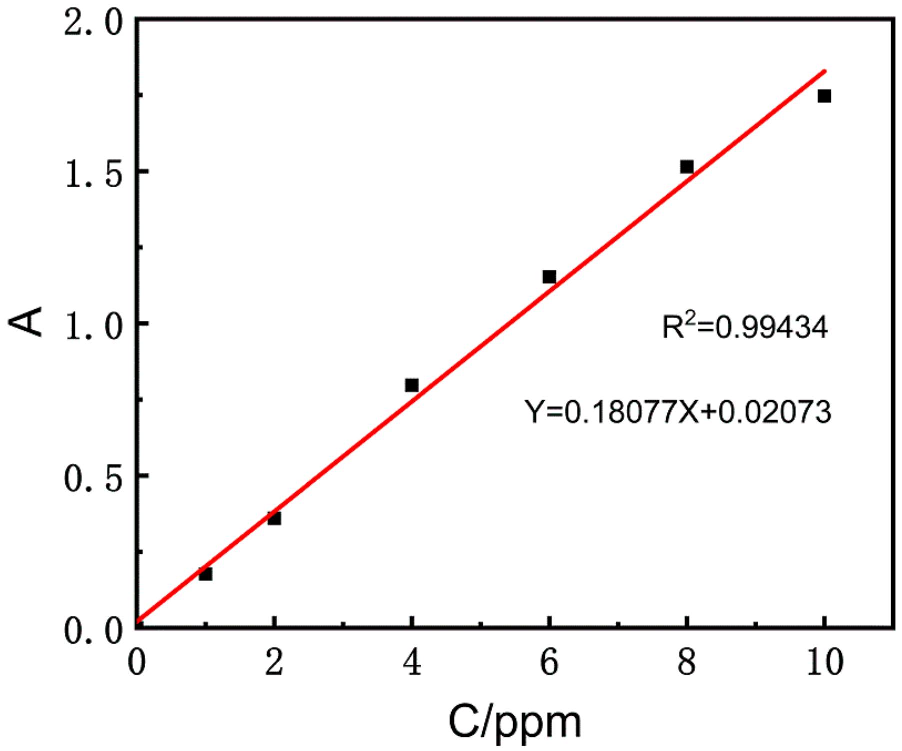

Figure S5. Standard curve for methylene blue solution; the horizontal coordinate is the concentration and the vertical coordinate is the absorbance.
